# Supplementary material for: Modeling glioblastoma heterogeneity as a dynamic network of cell states
Source: Mol Syst Biol. 2021 Sep 16;17(9):e10105. doi: 10.15252/msb.202010105 (PMC8444284; doi:10.15252/msb.202010105)
Supplement: Supplementary file 6 — Source Data for Figure 5 [file MSB-17-e10105-s004.zip › Figure5A_sourcedata/GSEA_3071/nosigdb_stateIII.GseaPreranked.1622795155562/LARSSON_STATE_5.html]

Details for gene set LARSSON\_STATE\_5[GSEA]

|  || Dataset | state33071 |
| Phenotype | NoPhenotypeAvailable |
| Upregulated in class | na\_pos |
| GeneSet | LARSSON\_STATE\_5 |
| Enrichment Score (ES) | 0.2570388 |
| Normalized Enrichment Score (NES) | 2.751504 |
| Nominal p-value | 0.0 |
| FDR q-value | 0.0 |
| FWER p-Value | 0.0 |
Table: GSEA Results Summary

  

Fig 1: Enrichment plot: LARSSON\_STATE\_5      
 Profile of the Running ES Score & Positions of GeneSet Members on the Rank Ordered List

  

| PROBE | GENE SYMBOL | GENE\_TITLE | RANK IN GENE LIST | RANK METRIC SCORE | RUNNING ES | CORE ENRICHMENT || 1 | RPLP0 |  |  | 6 | 0.633 | 0.0195 | Yes |
| 2 | RPL6 |  |  | 16 | 0.555 | 0.0355 | Yes |
| 3 | RPL37 |  |  | 20 | 0.526 | 0.0522 | Yes |
| 4 | RPL22 |  |  | 22 | 0.520 | 0.0692 | Yes |
| 5 | RPLP1 |  |  | 25 | 0.514 | 0.0858 | Yes |
| 6 | RPLP2 |  |  | 36 | 0.489 | 0.0994 | Yes |
| 7 | RPL18A |  |  | 58 | 0.453 | 0.1091 | Yes |
| 8 | RPL37A |  |  | 64 | 0.448 | 0.1226 | Yes |
| 9 | ZFAS1 |  |  | 65 | 0.447 | 0.1375 | Yes |
| 10 | RPL30 |  |  | 71 | 0.442 | 0.1509 | Yes |
| 11 | RPS28 |  |  | 73 | 0.440 | 0.1652 | Yes |
| 12 | RPL11 |  |  | 76 | 0.436 | 0.1792 | Yes |
| 13 | RPS19 |  |  | 84 | 0.427 | 0.1915 | Yes |
| 14 | RPL34 |  |  | 105 | 0.407 | 0.1999 | Yes |
| 15 | PABPC1 |  |  | 123 | 0.394 | 0.2085 | Yes |
| 16 | RPS27 |  |  | 132 | 0.388 | 0.2194 | Yes |
| 17 | TMSB10 |  |  | 148 | 0.376 | 0.2280 | Yes |
| 18 | RPL13 |  |  | 152 | 0.372 | 0.2396 | Yes |
| 19 | PPP1R14B |  |  | 174 | 0.359 | 0.2460 | Yes |
| 20 | RPL18 |  |  | 178 | 0.355 | 0.2570 | Yes |
| 21 | NDUFC1 |  |  | 284 | 0.296 | 0.2397 | No |
| 22 | RPL10 |  |  | 285 | 0.296 | 0.2495 | No |
| 23 | SARS |  |  | 367 | 0.270 | 0.2375 | No |
| 24 | DNM1 |  |  | 576 | -0.258 | 0.1923 | No |
| 25 | EPRS |  |  | 805 | -0.270 | 0.1422 | No |
| 26 | FAF2 |  |  | 907 | -0.274 | 0.1252 | No |
| 27 | HELLS |  |  | 970 | -0.278 | 0.1184 | No |
| 28 | TMEM59 |  |  | 1010 | -0.280 | 0.1176 | No |
| 29 | CD46 |  |  | 1039 | -0.281 | 0.1197 | No |
| 30 | TFPI2 |  |  | 1128 | -0.286 | 0.1064 | No |
| 31 | LAPTM4A |  |  | 1391 | -0.299 | 0.0486 | No |
| 32 | NKTR |  |  | 1552 | -0.309 | 0.0175 | No |
| 33 | CDKN1A |  |  | 1631 | -0.314 | 0.0077 | No |
| 34 | SRPX |  |  | 1747 | -0.322 | -0.0114 | No |
| 35 | PRH1 |  |  | 1783 | -0.324 | -0.0097 | No |
| 36 | ADAM9 |  |  | 1870 | -0.328 | -0.0210 | No |
| 37 | UBE2C |  |  | 1942 | -0.332 | -0.0284 | No |
| 38 | TIMP1 |  |  | 2038 | -0.337 | -0.0417 | No |
| 39 | LGALS3BP |  |  | 2152 | -0.345 | -0.0595 | No |
| 40 | CANX |  |  | 2209 | -0.349 | -0.0624 | No |
| 41 | HMOX1 |  |  | 2415 | -0.366 | -0.1033 | No |
| 42 | DYNC1H1 |  |  | 2580 | -0.380 | -0.1331 | No |
| 43 | SOX4 |  |  | 2595 | -0.381 | -0.1241 | No |
| 44 | PNISR |  |  | 2607 | -0.383 | -0.1142 | No |
| 45 | HMGCR |  |  | 2673 | -0.388 | -0.1181 | No |
| 46 | SMC4 |  |  | 2872 | -0.405 | -0.1559 | No |
| 47 | CCND2 |  |  | 3037 | -0.425 | -0.1842 | No |
| 48 | PDIA4 |  |  | 3130 | -0.435 | -0.1936 | No |
| 49 | PTPRG |  |  | 3246 | -0.451 | -0.2084 | No |
| 50 | MCAM |  |  | 3353 | -0.472 | -0.2201 | No |
| 51 | PCBP4 |  |  | 3381 | -0.476 | -0.2113 | No |
| 52 | ADGRV1 |  |  | 3529 | -0.520 | -0.2321 | No |
| 53 | DKK1 |  |  | 3556 | -0.530 | -0.2212 | No |
| 54 | VMP1 |  |  | 3570 | -0.536 | -0.2068 | No |
| 55 | RPN2 |  |  | 3581 | -0.539 | -0.1915 | No |
| 56 | RRBP1 |  |  | 3595 | -0.545 | -0.1768 | No |
| 57 | SQLE |  |  | 3606 | -0.553 | -0.1610 | No |
| 58 | CD9 |  |  | 3634 | -0.567 | -0.1491 | No |
| 59 | APLP2 |  |  | 3693 | -0.610 | -0.1439 | No |
| 60 | CENPE |  |  | 3711 | -0.625 | -0.1275 | No |
| 61 | APP |  |  | 3779 | -0.710 | -0.1213 | No |
| 62 | KCNQ1OT1 |  |  | 3866 | -0.885 | -0.1141 | No |
| 63 | CTGF |  |  | 3901 | -1.185 | -0.0836 | No |
| 64 | SNTG1 |  |  | 3909 | -1.316 | -0.0417 | No |
| 65 | IGFBP2 |  |  | 3917 | -1.404 | 0.0031 | No |
Table: GSEA details [plain text format]

  

Fig 2: LARSSON\_STATE\_5: Random ES distribution      
 Gene set null distribution of ES for **LARSSON\_STATE\_5**
